# Current Methods, Common Practices, and Perspectives in Tracking and Monitoring Bioinoculants in Soil

**DOI:** 10.3389/fmicb.2021.698491

**Published:** 2021-08-31

**Authors:** Andrea Manfredini, Eligio Malusà, Corrado Costa, Federico Pallottino, Stefano Mocali, Flavia Pinzari, Loredana Canfora

**Affiliations:** ^1^Council for Agricultural Research and Economics, Research Centre for Agriculture and Environment, Rome, Italy; ^2^National Research Institute of Horticulture, Skierniewice, Poland; ^3^Council for Agricultural Research and Economics, Research Centre for Viticulture and Enology, Conegliano, Italy; ^4^Council for Agricultural Research and Analysis of the Agricultural Economy, Research Centre for Engineering and Agro-Food Processing, Monterotondo, Italy; ^5^Institute for Biological Systems, Council of National Research of Italy (CNR), Rome, Italy; ^6^Life Sciences Department, Natural History Museum, London, United Kingdom

**Keywords:** soil, detection, microbial inoculants, bacteria, fungi, biofertilisers, biopesticides, microorganisms

## Abstract

Microorganisms promised to lead the bio-based revolution for a more sustainable agriculture. Beneficial microorganisms could be a valid alternative to the use of chemical fertilizers or pesticides. However, the increasing use of microbial inoculants is also raising several questions about their efficacy and their effects on the autochthonous soil microorganisms. There are two major issues on the application of bioinoculants to soil: (i) their detection in soil, and the analysis of their persistence and fate; (ii) the monitoring of the impact of the introduced bioinoculant on native soil microbial communities. This review explores the strategies and methods that can be applied to the detection of microbial inoculants and to soil monitoring. The discussion includes a comprehensive critical assessment of the available tools, based on morpho-phenological, molecular, and microscopic analyses. The prospects for future development of protocols for regulatory or commercial purposes are also discussed, underlining the need for a multi-method (polyphasic) approach to ensure the necessary level of discrimination required to track and monitor bioinoculants in soil.

## Introduction

Microbial-based products (also named bioinoculants, biofertilisers, and biopesticides) used to support plant nutrition and protection from abiotic and biotic stress have received considerable attention in the last decades by researchers, manufacturers and farmers, particularly because they help to reduce the use of chemicals in agriculture (Adesemoye et al., 2009; Lacey et al., 2015; Bargaz et al., 2018; Harman and Uphoff, 2019; Singh and Prabha, 2019; Basu et al., 2021). However, the inoculation of the soil with beneficial microorganisms may affect its native microbial populations (Trabelsi and Mhamdi, 2013; Mawarda et al., 2020); with effects that depend on the soil chemical and physical characteristics and the environmental conditions (i.e., climate, agronomic practices, and cropping systems, etc.). Furthermore, considering the pivotal role of soil microbial diversity for life-supporting functions, changes occurring to the soil microbial structure after applying microbial-based formulations may affect the overall soil quality and fertility with varying effects (Trabelsi and Mhamdi, 2013), which can impact crop productivity and quality.

On the other hand, manufacturers are interested in understanding the interactions between the bioinocula and the native soil microbiota since it can improve their application methods and efficacy. Moreover, particularly in the case of biopesticides, the knowledge of their effects on soil microbial diversity and composition must comply with ecotoxicological standards during the registration process (e.g., European Union Regulation 1107/2009). In this framework, which encompasses scientific, commercial and regulatory aspects, the development of tools to monitor the introduced microbial species becomes of paramount importance, particularly to assure a correct risk assessment in relation to the environment and human health (Sessitsch et al., 2019; Mitter et al., 2021). The monitoring tools should support two critical aspects of microbial inocula application: (i) the bioinoculant detection in the soil to evaluate its persistence and fate; (ii) the assessment of the impact of the introduced bioinoculant species on native soil microbial communities. Evaluating the effects on native soil microbial communities and detect the inoculants once applied in the soil can add a piece in the complex puzzle of biofertilizer development.

The present review focuses on the methods so far applied or with future potential for tracking the bioinoculants in soil, and evaluating their impact on the local microbial communities. The aim is to provide critical and comprehensive analysis of the tools currently available, and also a bibliometric screening of the literature dealing with detection and monitoring of microbial inocula. A description and comparison of the methods based on morpho-phenological, molecular and microscopic approaches is then provided. The prospects for the future development of protocols suitable for regulatory or commercial purposes are also discussed. A multi-faced approach capable of assuring the necessary level of discrimination that is required for tracking and monitoring bioinocula and biopesticides in the soil is then suggested.

## Trends in Soil Microbial Inoculants Detection and Monitoring: a Science Mapping Approach

A science mapping analysis of published research papers was used to appraise the research trends on microbial inoculant detection and monitoring in the soil in 1991–2020. The following keywords and strings were used to retrieve the relevant publications from the SCOPUS database, which was consulted on October 12th, 2020: detection, tracking, traceability, monitoring, inoculants (OR bioproducts OR biofertiliser OR consortia OR microorganisms OR bioinocula), soil, technique (OR tool OR method) in the combined fields of “title,” “abstract,” and “keywords.”

The software VOSviewer, version 1.6.5.0 (freely available at www.vosviewer.com), was used to create bibliometric maps based on the retrieved publications and to conduct a general quantitative analysis. Before starting the analysis, a thesaurus file was created to ensure consistency for different term spelling and synonyms. Term maps were produced following the method used by van Eck and Waltman (2010). The EndNote file of this search is attached as Supplementary Information Data 1 and VosViewerMap, and Network files necessary to navigate the maps with labels are available as Supplementary Information Data 2, 3 respectively.

**FIGURE 1:**
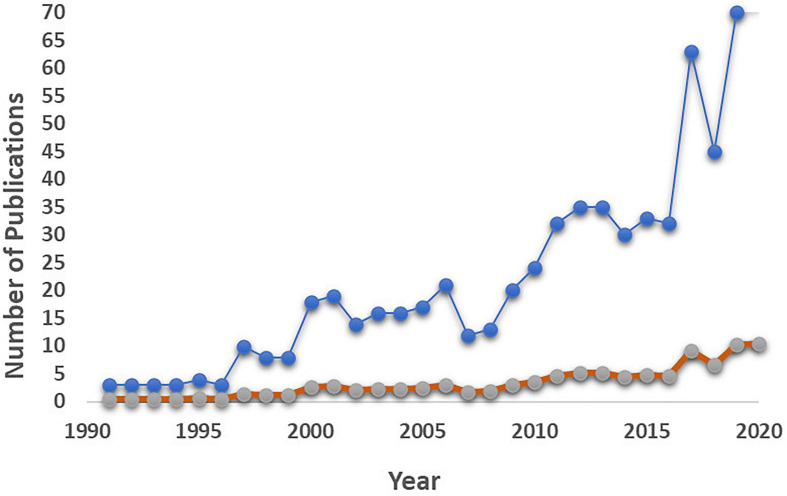
Trend of publications on soil microbial inoculants tracking and monitoring in the period from 1991 to 2020. The blue line represents the number of publications per year; the orange line represents the frequencies of publications per year.

A total number of 681 scientific publications were retrieved, providing in total 17,617 terms. Only the terms occurring at least eight times in the combined fields per publication were extracted from the 681 retrieved publications. Figure 1 shows the yearly total number and frequencies of publications in the concerned period. It emerged that during the first 5 years, only 1 to 3 articles were published yearly on these subjects, while from the beginning of the century, the interest in the topic, and thus the number of publications, increased, generally in parallel to the research on microbial bioinocula (Santos et al., 2019; Canfora et al., 2021). Almost 70% of the retrieved publications (474) were research papers, while the remaining 207 included both reviews (114) and books or chapters. Interestingly, the countries traditionally concerned with soil microbiology studies and the introduction of microbial inocula in the agronomical practice, also having relevant legislation in place, resulted at the top of the list: the European Union (21.64%, with Germany accounting alone for the highest percentage – 6.31%), India (11.32%), United States of America (9.92%), China (7.52), and United Kingdom (5.41).

**FIGURE 2:**
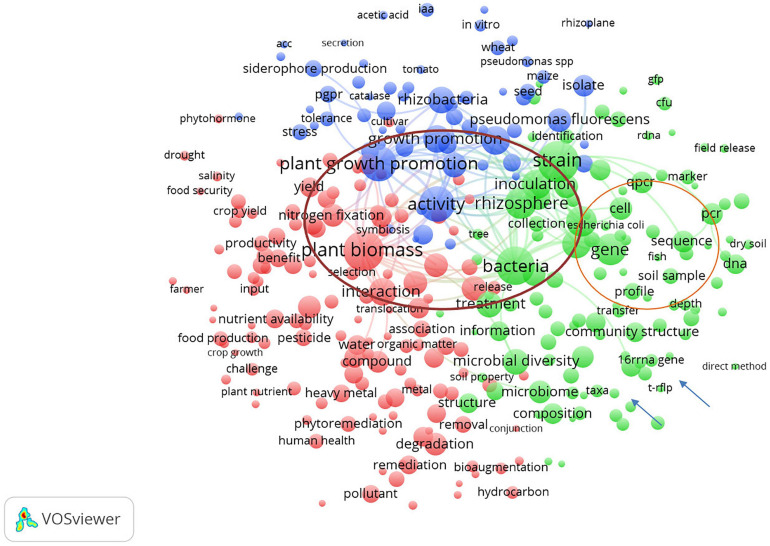
The terms clustering map based on the analysis of publications concerned with soil microbial inoculants tracking and monitoring retrieved from Scopus database from the period 1991–2020. Red, blue, green colors represent the terms belonging to different clusters. The dot size of each term is based on the number of its occurrence. The connecting lines indicate the 100 strongest co-occurrence links between terms.

Term maps showing closeness among terms were calculated based on terms co-occurrence within the same publication, according to Costa et al. (2019). Three clusters emerged from the analysis, discriminating methods-based terms (e.g., qPCR, marker, PCR, sequence, and T-RFLP; green circles), microorganism-related terms (e.g., *Pseudomonas fluorescens*, *Rhizobacteria*, plant growth promoting bacteria, and activity; blue circles), and use-related terms (e.g., plant biomass, nutrient availability, remediation, and pollutant; red dots; Figure 2). A number of terms formed a bridge between clusters (i.e., strain, inoculation, bacteria, and rhizosphere), while others appeared quite scattered (i.e., drought, phytohormone, farmer, human health, and field release).

It is noteworthy that the term “field release” and “farmer” are set apart from each other, which could suggest a limited connection between research and field application of bioinocula or a limited number of long-term studies under field conditions assessing the impact caused by or persistence of microbial inoculants on the soil ecosystem and microbiome as also pointed out recently elsewhere (Canfora et al., 2021).

It shall be highlighted that the term “detection” appeared first in 1991 (Pickup, 1991), and a review on the microbiological and molecular techniques for monitoring the genetically modified and unmodified microorganisms in the soil appeared in 1998 (Van Elsas et al., 1998), pointing out questions about risk assessment concerning the release of cells in the soil environment. However, terms like “regulation” and “legislation,” which should be widely expected, were missing from the analyzed dataset of publications. On the other hand, terms related to results of soil inoculation with bioinocula were associated only with beneficial effects.

**FIGURE 3:**
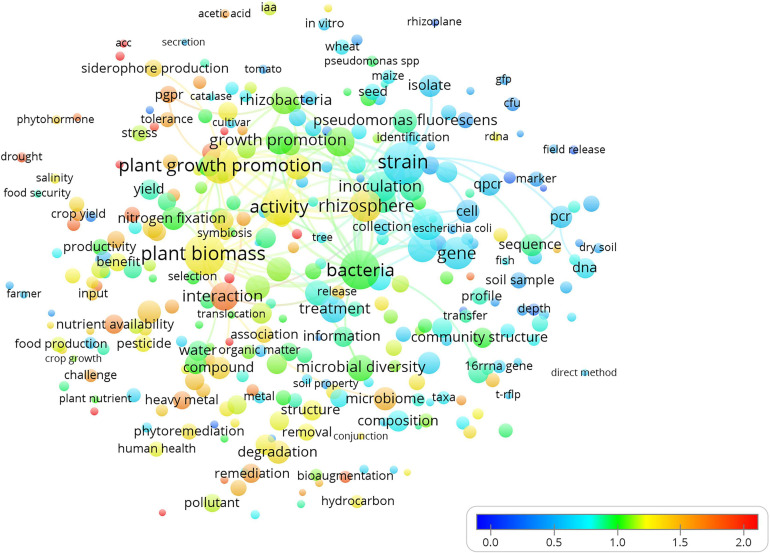
Term map based indexed per citation rate based on all the Scopus soil microbial inoculants tracking and monitoring publications/citations. The different colors represent the normalized citation rate scale. The size of each term is based on the number of its occurrence. The connecting lines indicate the 100 strongest co-occurrence links between terms.

**FIGURE 4:**
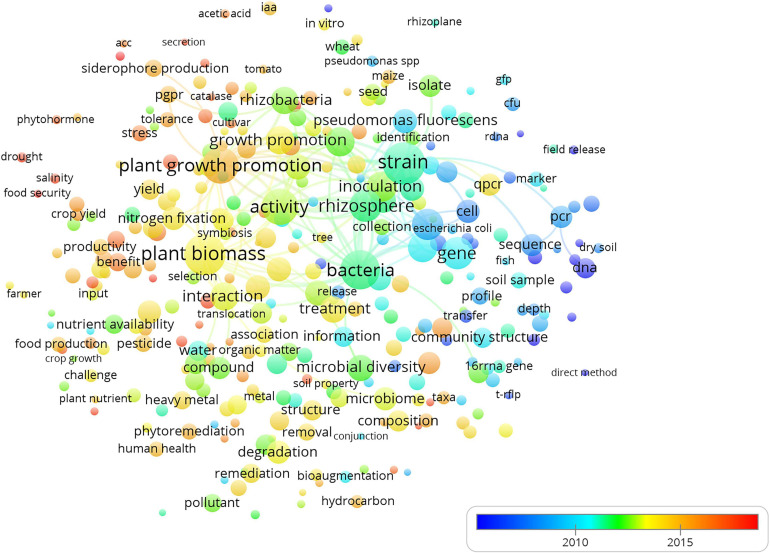
Term map created according to publication year on soil microbial inoculant detection and monitoring based on the Scopus database. The time scale is represented by different colors. The dot size of each term is based on the number of its occurrence. The connecting lines indicate the 100 strongest co-occurrence links between terms.

The term map created according to the publication year (Figure 3) clearly showed the evolution and trends in research during the last decade. The attention toward the study of the interaction between native soil microbial communities and bioinocula represented by terms such as “interaction,” “plant growth promotion,” and “activity” has marked the new trend in the detection and monitoring of microbial soil inoculants. The increased sensibility during the last decade with respect to the environmental impact of bioinocula also emerged from the map representing the scientific impact of the subjects addressed by researchers over the years (Figure 4).

A striking result of the analysis concerned the unbalance of studies with terms associated with bacteria, which overrated those dedicated to fungi. This outcome confirmed another recent metadata analysis, which found that among 108 studies addressing the use of microbial inoculant in soil, only 22 were dealing with inoculated fungi and two the co-inoculation of bacteria and fungi, while the remaining concerned bacteria (Mawarda et al., 2020). Even though both groups of microorganisms have a long history of field application as in the case of mycorrhizal fungi (Wamberg et al., 2003; Bardi and Malusá, 2012; Malusá et al., 2016) or N-fixing bacteria (Lucy et al., 2004; Naseer et al., 2019), the easiness and/or wide availability of strains inducing growth promotion or protection among the bacteria could have been accounted for this result, also confirming the conclusions of other analyses (Canfora et al., 2021).

The most recurrent methods for detection and monitoring in the publications retrieved were based on PCR techniques. The terms PCR/qPCR showed the highest occurrence (150 items), while culture-dependent and fingerprinting [e.g., terminal-restriction fragment length polymorphism (TRFLP), Denaturing Gradient Gel Electrophoresis (DGGE)] methods occurred only about 50 times each. Mawarda et al. (2020), by using a literature search-mapping approach, reported that 72% of studies addressing the impact of bioinocula on resident microbial communities used profiling methods including TRFLP, and 4% used quantitative PCR targeting particular taxonomic or functional groups. The use of a single methodology for the detection and monitoring of the inoculated strain was already argued by Van Elsas et al. (1998). They recommended a polyphasic approach, combining culture-dependent and culture-independent methods, to evaluate the dynamics of non-native microorganisms introduced in the soil environment and their interaction with the autochthonous microbiome to understand their survival and performance. However, the current and previous bibliometric analyses showed that a monophasic approach had been generally used to address these goals. The effort expressed by this review in presenting and discussing comprehensively different available and underutilized or perspective methods for detection and monitoring of bioinoculants also aimed at fostering a better understanding of the opportunities and risks associated with the use of bioinocula.

## What to Keep in Mind When Monitoring the Impact of Microbial Inoculants in Soil Interactions Between Bioinocula and Autochthonous Soil Microorganisms

The mechanisms underlying the fate and persistence of bioinoculants in soil can result from the sum of multiple variables and therefore be difficult to understand and predict. The introduction of any microbial inoculant to the soil should be considered as a disturbance to native microbiota. In ecology, the term “disturbance” usually refers to causal events that may directly or indirectly impact the environment/community and may occur at different spatial and temporal scales with different frequencies, intensities, and extents (Shade et al., 2012). A community’s response to disturbance is defined as “stability” that comprises both “resistance” and “resilience.” The scientific literature includes many definitions of stability, resistance and resilience, and a complete examination of these definitions is available elsewhere. However, in this paper, the term “resistance” is defined as the degree to which a community is insensitive to a disturbance, and “resilience” is the rate at which a community can return to the original state after a disturbance. Many studies have investigated the resilience of microbial communities to various disturbances, including human activities such as land use and agricultural practices (Mocali et al., 2015; Morriën et al., 2017) but also natural disturbances such as fire or freeze-thaw and desiccation (Shade et al., 2012; Griffiths and Philippot, 2013). However, to the best of our knowledge, the impact of microbial inoculation on the soil microbiome has been poorly addressed so far. In particular, the extent to which these changes affect soil ecosystem functioning remains largely unknown (Ray et al., 2020).

Bioinocula releases into soil often result in transient loads of the microbial strain/s that generally fade away with time (Bankhead et al., 2004), even though it has been shown that an entomopathogenic fungal strain was detected up to 15 years after the application (Enkerli et al., 2004; Mayerhofer et al., 2015). Given the supposed transitory survival of bioinocula, many scientists and practitioners assume that soil microbial inoculants would have negligible effects on the autochthonous soil microbial communities (Mawarda et al., 2020). However, the quick disappearance of inoculum in the soil does not necessarily imply a lack of long-lasting changes in the soil resident community. For example, *Rhizobium* inoculation to promote soybean productivity significantly influenced the bacterial community in the crop rhizosphere (Zhong et al., 2019) and the fungal community (Xu et al., 2020). Furthermore, the introduction of non-pathogenic *Escherichia coli* into soil shifted the niche structure. It increased the niche breadth of resident bacterial communities, leading to changes in significant soil bacterial genera’s relative abundances, such as *Bacillus*, *Pseudomonas*, *Burkholderia*, and *Bradyrhizobium* (Mallon et al., 2018). On the other hand, it is logical to assume that persisting microbial inoculants will have a more prolonged impact than short-lived inoculants. For instance, long-lasting changes were reported in microbial composition with the inoculant *Bacillus amyloliquefaciens* NJN-6, which showed relatively stable abundance (between 2.5 and 3.0 log copies of 16S rRNA gene/gram of soil) within 3 years after application (Fu et al., 2017). However, it remained unclear whether the measured changes were due to direct effects from the inoculant or indirect effect, for instance, because of nutrients released from dead inoculant cells or the inoculant-root interactions described for other bioinoculants (Castro-Sowinski et al., 2007; Hartmann et al., 2009).

The survival of microbial inoculants in soil depends on the soil native microbiota: it is high when the autochthonous microbial community’s diversity is low and vice versa (Van Elsas et al., 2012; Mallon et al., 2015). However, the importance of evaluating the impact of inoculation in a temporal context should be kept in mind since the effects might change over time. For example, most of the studies addressing the impact of microbial inoculants on soil native communities measured the effects only over a short period after inoculation, usually within the vegetative season (Mawarda et al., 2020). In most cases, the inoculation led to changes in the structure of the resident communities, but it remains unclear whether the impact could persist for a more extended time, thus indicating the actual resilience capacity after the disturbance (Xing et al., 2020). However, some studies have shown a longer-term impact on resident rhizobacteria diversity due to residual effects of antibiotic-producing PGPR *P. fluorescens* F113Rif (Walsh et al., 2003) or on the community structure of some specific bacterial groups (Robleto et al., 1998). Moreover, the method applied for the assessment could differently represent the impact of the bioinocula. For example, the T-RFLP-based methodology and also the quantification of the gene copy numbers of a specific set of fungal genes showed that the application of two different species of entomopathogenic fungi did not alter the composition of fungal communities considerably neither after the first application nor at the end of the second season in comparison to untreated soils (Tartanus et al., 2021) but appeared to modify the bacterial community. Remarkably, even unsuccessful application of microbial inoculants might still leave a legacy effect on the resident community (Mallon et al., 2015; Xing et al., 2020). Therefore, although largely unexplored, this issue might have implications in areas where microbial invasions are sought, such as on the efficacy of probiotic, biocontrol, and biofertiliser agents, because the legacy of past invasion attempts may alter the present community structure, functioning, and, ultimately, mechanisms of resistance.

Considering the role of the resident soil microbiome for an effective use of microbial inoculants, it is quite surprising that the term “resilience” was not evidenced by our literature survey and science mapping approach. It confirms that this perspective is still largely overlooked, thus suggesting the need for an effort to evaluate the impact of inoculants on soil native community resilience, especially from a functional perspective. A broad range of “omic” approaches (metagenomics, metatranscriptomics, metabolomics, and metaproteomics) applied in long-term experiments under field conditions would allow an in-depth assessment. An example of such effort is represented by the EXCALIBUR project^1^, aiming to deepen our understanding of the mechanisms underlying the soil microbiome changes composition and functioning upon bioinocula application (Allison and Martiny, 2009).

## Good Field Practices to Track Soil Microbial Inoculants: the Sampling Approach Comes First

The complex web of soil microorganisms represents the primary biodiversity source on Earth (Shoemaker et al., 2017). Research in soil microbial ecology has revealed the tremendous diversity and complexity of microbial communities across space and time in the soil (Green and Bohannan, 2006). The difficulties associated with the soil matrix (uneven physical and chemical composition), which require a specific effort when studying microbial diversity, are even more challenging when tracking or monitoring a bioinoculum is concerned (Nannipieri et al., 2019).

Therefore, the sampling method used for tracing and monitoring the fate of a microbial inoculant in the soil represents the most critical step of the process, which shall be designed to assure the quality and the robustness of the data generated.

The sampling methodology shall consider whether bulk or rhizospheric soil (or almost rhizospheric, i.e., in the root zone) soil is best suited to assess the strain fate. For strains used as biofertilisers, the soil near the roots is considered more suitable because the plant-microbe interactions (Bhattacharjee et al., 2008; Hartmann et al., 2009) can enhance the bioinoculant survival and root colonization. Inoculants used as biopesticides, particularly those based on fungi able to behave saprophytically (e.g., *Beauveria bassiana*, *Trichoderma* spp., etc.), can also be monitored by sampling either the bulk soil (Enkerli et al., 2004; Kessler et al., 2004; Dolci et al., 2006; Mayerhofer et al., 2015), or the soil near the root system in case of poly-annual/perennial crops (Canfora et al., 2016; Tartanus et al., 2021).

Sampling the bulk or soil near the root zone will dictate the depth at which the soil has to be collected. However, the soil texture and composition should also be taken into consideration in defining the sampling depth due to the possible effect of oxygen (air) availability and organic matter content on bioinoculant persistence (Walstad et al., 1970; Studdert and Kaya, 1990; Vänninen et al., 2000; Weyman-Kaczmarkowa and Pȩdziwilk, 2000; Kessler et al., 2003; Rousk and Bååth, 2007; Fàtu et al., 2015). The frequency of sampling is a variable that can be affected by several factors, including the formulation of the bioinoculum, the crop duration, the strain growth potential, and the pedo-climatic conditions of the site (Horaczek and Viernstein, 2004; Malusá et al., 2020). All of them are quite intuitive to understand; however, a proper sampling strategy cannot disregard them to avoid reducing of the validity of the analytical results. The number of samples and sub-samples to be collected for each individual analysis is generally constrained by the resources available for the analytical determination. However, the minimum number of samples should comply with the classical soil microbiology methods (Bloem et al., 2009; Elsas et al., 2019).

Examples of sampling strategies to monitor the persistence and fate of bioinocula in soil are available, particularly for biopesticides. This is mainly due to the registration requirements because of concerns about non-target organisms’ risks (e.g., EU Regulation 1107/2009, US EPA Act – 7 U.S.C. §136 et seq. 1996; Pelaez et al., 2013). For example, a randomized block design with 4 replicates, sampling the soil near the root zone at a depth of 0–20 cm, with each soil sample composed by 25 points randomly distributed within each treatment was used to follow the persistence of formulations made with two *Beauveria spp.* in strawberries (Canfora et al., 2016; Tartanus et al., 2021). The persistence and fate of five *B. brongniartii* strains in the soil for 2 years were monitored collecting 12 soil samples from a depth of 5–20 cm in eight apple orchards (Dolci et al., 2006). However, the increasing awareness about the implications derived from the field application of microorganisms could also foster the introduction of similar requirements for biofertilisers [EFSA project GP/EFSA/ENCO/2020/02 on “Evaluating the impact on/by environmental microbiomes (plants, wildlife, and soil) in assessments under EFSA’s remit”].

Soil handling also requires a specific procedure after sampling, particularly when molecular techniques are planned to be used for the analysis. It must be kept in a sterile plastic bag or tube DNase and RNase free with minimal manipulation, placed in a cooler with ice or ice bags and, once in the laboratory, it shall be stored immediately at 4°C or frozen in case of prolonged storage periods.

## Overview of the Methodological Tools to Detect and Monitor Bioinocula in Soil: Challenges, Limitations, and Controversial Issues

Earlier detection approaches from culture-dependent tools, such as direct microscopic examination (Stefani et al., 2015), plate profiling (Davis et al., 2005; Eichorst et al., 2007; Bloem et al., 2009), and FISH (Fluorescent *in situ* Hybridization; Cerqueira et al., 2008; Schmidt and Eickhorst, 2014), have provided essential insights into detection and localization of target microbial species in soil. These approaches led the way to culture-independent tools addressing the analysis of target microbial species of bioinocula and evaluating the bioinocula effect on microbial communities’ structure and diversity. The rapid development of DNA and RNA-based analytical methods has offered new opportunities to monitor microbial inoculants survival and interactions within a specific soil community. Indeed, a high degree of resolution is fundamental to evaluate the success or failure of bacteria or fungi inoculation, tracing the “introduced DNA” in a mixture of genomes from thousands of different organisms. Culture-independent methods have also effectively characterized the soil microbial assemblages in space and time, evaluating their functional and trophic interactions (references herein included). Recent research in the “omic” era has expanded our knowledge and understanding of microbial community assembly but tracing the bioinocula in the soil is still not a straightforward task. A plethora of methods have been developed to enable inventories of microbial species composition and a good understanding of dynamics and processes of biodiversity (Philippot et al., 2012; Nemergut et al., 2013; Cristescu, 2014) in bulk or rhizospheric soil, but generally are not suitable to follow the fate of a single species.

However, any method utilized for tracking inoculants introduced in the soil presents some advantages and disadvantage, which we try to point out to highlight possible procedures implementing the multi-phasic approach mentioned above.

### Microscopy-Based Techniques: Challenges and Opportunities

Given the wide variability of microbial species, the observation, identification, and enumeration of microorganisms not as pure isolates but embedded in complex matrices, such as plant cell tissues, biofilms, soil, compost, and mineral matrices, require the use of rigorous procedures. Different species or strains may differ in morphology, nutrition, physiology, reproduction and growth, metabolism, pathogenesis, antigenicity, and genetic properties (Moore et al., 2010). Techniques developed to morphologically resolve and trace individual species or even particular strains of microorganisms in a chemically and physically complex system generally exploit one or more organisms’ properties, combining their resolving power to achieve maximum discrimination. Morphology, including cell size, arrangement and shape, is not sufficient on its own to typify and trace individual species of microorganisms, primarily if they are embedded in complex matrices that are opaque to the source of observation (Moore et al., 2010).

Tracing individual species or strains of fungi or bacteria in environmental matrices leads back to the fundamental concepts of taxonomy (or biosystematics), which consists of four main parts: classification, nomenclature, identification and phylogeny, which is now an integral part of the classification process (Naranjo-Ortiz and Gabaldón, 2019). However, prokaryotic microorganisms vary widely in size, and their cells are often close to the resolution limits of the light microscope; also, when observing environmental samples, cells may become confused with particulate matter or be obscured by internal and external structures of larger cells, as is the case with animal and plant tissues (Johnson and Criss, 2013). Furthermore, it is often impossible to distinguish living cells from dead cells or cells from inanimate objects in environmental samples.

Compared to bacteria, fungi have larger cells that often display characters with a diagnostic value, but the systematics of fungi is complicated by intraspecific morphological and physiological variation and the limited number of morphological markers available (Naranjo-Ortiz and Gabaldón, 2019). Therefore, the use of microscopy to locate, identify and count individual microbial taxa is limited by the systematic weight of the characters used in the identification.

Fluorescence microscopy (FM) has represented a significant advance in tracking and localizing specific microorganisms in complex matrices. Using fluorescent dyes (fluorophores) and markers derived from them that indicate the presence of specific substances or cellular activities, it is possible to visualize microorganisms under the microscope and distinguish them from the background or dead cells in the same sample. These protocols combine membrane-permeable fluorescent dyes [i.e., SYTO9 and 4′,6-diamidino-2-phenylindole (DAPI)], which penetrate all the microbial cells in the sample, with membraneimpermeable fluorescent dyes, that instead are impermeable only to viable membranes (i.e., propidium iodide and SYTOX Green), and which therefore enter only the non-viable cells (Johnson and Criss, 2013). These techniques work with both fungi and bacteria, but some cells and resistant structures could require specific protocols, as well as with the complicated wall structure of many fungal taxa (Villa et al., 2009). Many fluorescent probes have been developed, which, when used in combination with the FM, allow various cellular parameters of microorganisms to be measured, thereby tracking their presence and activity. For example, fluorescence detection techniques have been widely used to map intracellular pH. Fluorescence lifetime imaging microscopy (FLIM) allow characterizing pH regulation mechanisms in different cellular compartments. Carboxy–(C–) SNAFL1, C–SNAFL2, fluorescein, and C–fluorescein are fluorescent compounds that allow real–time cellular response to pH changes in the medium, at different pH. They are widely used to visualize pH gradients in microbial biofilms (Lin et al., 2003). However, unless it is sufficient to generically track a cellular function or enzymatic activity to detect the presence of the microorganism of interest, chemical–only fluorescent markers do not have the resolution power required of a system capable of discriminating between different microorganism species. One of the limitations of FM and derived techniques is the spatial resolution, which is usually in the order of 30–100 nm (Huang et al., 2009).

The use of fluorescence *in situ* hybridization (FISH) techniques, with oligonucleotide probes that bind only to complementary nucleic acid sequences to target rRNA molecules, is a key method for detecting and sometimes identifying microorganisms in environmental samples. Probes can be designed for any gene or sequence within a gene to visualize target mRNA in microbial cells. FM combined with FISH can then be used to visualize the target microorganism in the sample where the fluorescent probe has bound. There are many FISH techniques with application–specific protocols and different fluorochrome combinations (Schimak et al., 2016). FISH is widely used in microbial ecology to identify microorganisms in complex matrices, although one of the best–known applications is the characterization of multispecies biofilms (Almstrand et al., 2013). With FISH, by using different colored probes for each species present in a sample, it is possible to localize their distribution and document their interaction by FM. Recently, multicolor FISH approaches have been developed using up to eight fluorophores with distinct spectral properties. The technique allowed to unambiguously discriminate the seven phylogenetically distinct microbial populations that were composing an artificial community (Lukumbuzya et al., 2019).

Oligonucleotide probes targeting ribosomal RNA (rRNA) are typically used to identify bacteria, being among the most conserved macromolecules in nature and found with a high copy number in every cell. Comparative analysis of rRNA sequences allows identifying short signature sequences that are unique to different groups of microorganisms. These signatures are used as targets for fluorochrome–labeled probes made of short complementary oligonucleotides (15–30 nucleotides). A comprehensive online resource for information on the identification of single microbial cells by FISH is available on the SILVA website^2^.

Raman micro-spectroscopy, a vibrational spectroscopy technique integrated with microscopy systems, is another microscopy-based technique suitable for tracking microorganisms in complex matrices that allow the chemical fingerprinting of individual eukaryotic organelles or bacterial cells. The chemical information derived from a Raman spectrum makes it possible to identify substances accumulated in cells, such as lipids, sugars, cytochromes, and pigments, within seconds (Gruber-Vodicka et al., 2011; Majed et al., 2012; Milucka et al., 2012). The incorporation of stable 13C, deuterium and 15N isotopes into target cells allows their detection by characteristic band shifts in Raman spectra (Wang et al., 2016). The combination of Raman micro-spectroscopy with FISH techniques allowed the authors to recognize *in situ*, in a microbial community, cells capable of degrading naphthalene and attributing them to an uncultured bacterial species (Huang et al., 2007).

Another spectroscopic technique associated with microscopy and capable of discriminating between different microorganisms’ species is the Fourier-Transformed Infra-Red spectrometry integrated with a microscope (FT-IR microspectroscopy). The technique is used to create a chemical fingerprint of the target microorganism, which is then used to discriminate it from other strains (Adiani et al., 2020). This technique was used to discriminate *E. coli* strains from different sources, and its efficiency was comparable with the BOX-PCR genomic fingerprinting method (Carlos et al., 2011). The bands in the FT-IR spectra that were responsible for the strain’s discrimination were in the region between 2,816 and 3,026 cm^–1^ wavenumbers, corresponding to fatty acids.

Discrimination between cells of different microbial species that coexist requires a spatial resolution that can only be achieved by electron microscopy (EM). However, the limited field view and small area visualized in EM images represent a limitation for the technique. To monitor larger areas of the sample by EM and understand specific patterns of microorganism distribution or assess their abundance, it is necessary to scan complete EM grids with a resolution that would require the collection of thousands of images. This limitation can be overcome by correlative microscopy, which combines fluorescence and EM (Verkade, 2008). FM is used to identify and localize features of interest, and EM is used to map their ultrastructure (Schwarz and Humbel, 2007). The development of increasingly advanced correlative microscopy systems goes hand in hand with software and computing power to handle complex sample coordinates and manage in parallel several microscope software (Agronskaia et al., 2008). Correlative microscopy initially combined optical or FM with transmission EM, but it is now available in integrated systems that combine different microscopy and spectroscopy types, making it possible to find and analyze the same points in a sample with nanometric precision. Wrede et al. (2008) used a scanning electron microscope in conjunction with an optical fluorescence system to study microbial mats from Cold Seep communities, simultaneously visualizing and distinguishing bacterial and archaeal microorganisms and their associated biogenic minerals. Instruments in which FM is correlated with scanning EM are often complemented by additional techniques that exploit the possibility of performing different measurements at the same point of interest in a sample, switching between instruments, or integrating multiple analysis methods in the same instrument, with software that manages coordinates and recognizes image patterns (Kommnick et al., 2019). For example, Liu et al. (2020) used confocal FM, time-of-flight secondary ion mass spectrometry, X-ray photoelectron spectroscopy, and scanning electron microscopy (SEM) in an integrated system to document, through imaging and both organic and inorganic chemical analysis, the interaction of *Brachypodium* roots with a strain of *Pseudomonas* spp., a typical plant growth-promoting soil bacterium.

For tracking microorganisms in environmental samples and tissues, such as roots and plant organs, *in situ* hybridization systems with nucleic acid-based metal probes allow the observation of samples directly with a scanning electron microscope (SEM-ISH) is of particular interest. The technique involves hybridizing target cells with gold- or platinum-labeled oligonucleotide or polynucleotide probes. It is, therefore, similar to FISH but allows observation directly at the resolution offered by scanning electron microscopes, without necessarily using correlative microscopy equipment. A comprehensive discussion of the technique was addressed by Kenzaka and Tani (2012), and effectively used to track bacteria hybridized with DNA-targeted polynucleotide probes increasing the method sensitivity by applying a signal amplification (Kenzaka et al., 2009). The SEM-ISH protocol proposed by Kenzaka and Tani (2012) allows detecting of cells with a low number of target DNA sequences, enabling to determine the spatial distribution of target cells in complex communities and on different materials. With SEM-ISH technique, SEM instruments can also provide phylogenetic or genetic information on target microbes in addition to information on the substrate on which they grow, both in terms of high-resolution imaging and chemical imaging (i.e., with backscattered electrons, that allow distinguishing objects with a different atomic number; Goldstein et al., 2018).

### Culture-Dependent Methods

Culture-dependent methods have several advantages as practical techniques to quantify bio-inoculants. They permit the detection of only viable cells and, therefore, inoculants that are competitive and persist over time. However, using these methods, detecting the inoculated strain from field samples is a difficult task (Pitkäranta et al., 2011; Al-Awadhi et al., 2013; Ngom and Liu, 2014). Culture-dependent methods cannot provide a comprehensive analysis of the endophytic ability of strains under unsterilized conditions, and epiphytes resistant to sterilizing agents could determine an overestimation of their counts (Kandel et al., 2017).

To fully understand the power and limitations of culture-dependent methods in monitoring specific bioinocula in soil, it is necessary to consider that although ongoing cultivation efforts continue to yield between 600 to 800 novel bacterial species per year (Overmann, 2013), global bacterial diversity ranges between 10^7^ and 10^9^ species (Curtis et al., 2002). The same situation applies to fungal species, which are estimated between 70,000 and 1.5 million (Feofilova, 2001), with only 5% of them described and cataloged (Guarro et al., 1999). Notwithstanding the abundance of microbial species in soil, more than 99% cannot be cultured by traditional techniques. Therefore, the culturable species are not representative of the total phylogenetic diversity. Nevertheless, considering that commercial or potential bioinocula are produced by fermentation (i.e., must be culturable), the culture-dependent methods are technically suitable for bioinocula monitoring or tracking (Table 1). Indeed, since the beginning of microbiology, culture-based methods have been used to assess microorganisms’ viability. However, bacterial cells that cannot grow on routinely used laboratory media were considered to be dead and described as “viable” but non- “culturable” (Xu et al., 1982; Bodor et al., 2020).

**TABLE 1:** Culture-dependent approach used to monitor soil inoculum.

Strains	Experimental conditions	Microbial media	Plant substrate	Results	References
*Pseudomonas* sp. G1Dc10 *Paenibacillus* sp. G3Ac9 *Sphingomonas azotifigens* DSMZ18530	Gnotobiotic conditions in a controlled environment chamber (16-h light/8-h dark, 18–23°C)	TY agar	Modified Evans medium supplemented with 8% agar	Colonization density in the rhizoplane and the leaves were about 9 and 4 log10 CFU/g, respectively. Colonization was more abundant in the rhizoplane than in plant tissues.	Castanheira et al., 2017
*Pseudomonas* sp. VM1449 *Pseudomonas* sp. VM1450 *Pseudomonas* sp. VM1453	Pots (16-h light/8-h dark, 20–25°C)	PCA containing 100 μg/mL kanamycin	Sterilized compost/vermiculite (3:1 ratio)	The three bacterial strains showed differently colonization behavior (CFU/g) for the rhizosphere, and interior root tissues stem or leaves	Germaine et al., 2004
*Burkholderia* sp. WPB *Rhizobium tropici* PTD1 *Rahnella* sp. WP5	Axenic conditions in a growth chamber	MG/L with 100 μg/mL of gentamycin and carbenicillin	N-free MS agar	Higher endophyte populations (CFU/g) were observed in the roots when compared with the stem and leaves	Kandel et al., 2015
*Azotobacter chroococcum* HKN-5 *Bacillus megaterium* HKP-2 *Bacillus mucilaginous* HKK-2 *Glomus mosseae Glomus intraradice*	Pots in a greenhouse (20 ± 4°C; 87 days)	Specific media for N-fixing bacteria, P solubiliser and K solubiliser	Soil (pH 5.46, organic matter 1.08%, total N 0.062%, total K 7,408 mg/kg, total P 1,090 mg/kg)	The population size of the inoculated rhizobacteria varied following the levels of fertilization and AMF colonization in the rhizosphere	Wu et al., 2005
*Azotobacter chroococcum Bacillus megaterium Bacillus mucilaginous Glomus fasciculatum Glomus mosseae*	Greenhouse (21 ± 5°C; 45 days)	Differentiating media for N fixing bacteria, P solubiliser and K solubiliser	Sterilized soil (pH 7.32, EC 0.14 dS/m, total C 1.92%, total N, 0.19%, total K 2,063 ppm)	Root colonization by AMF was increased in the presence of bacterial consortium application in comparison to individual inoculation treatments	Khalid et al., 2017
*Azotobacter* strain ST3 *Azotobacter* strain ST6 *Azotobacter* strain ST9 *Azotobacter* strain ST17 *Azotobacter* strain ST24	Pothouse; sampling at 30, 60, and 90 days	Nutrient agar	Four different unsterilized saline soil	Survival of inoculated strains increased up to 60 days of sampling	Chaudhary et al., 2013
*Azotobacter chroococcum* 76A	Greenhouse (10 cm plastic pots)	LG agar	Pure peat moss under salt stress	The bacterial strain was able to grow in the rhizosphere of tomato plants under abiotic stress conditions increasing of 1 Log	Van Oosten et al., 2018
*Azotobacter chroococcum* Mac 27L	Pots; sampling after 30 and 60 days of growth	Burks medium plates with and without X-gal	Unsterilsed soil	The bacterial strain was able to survive in the rhizoplane of *Brassica campestris* up to 30 days after sowing	Solanki and Garg, 2014
*Azotobacter chroococcum* AZ1 *Azotobacter chroococcum* AZ2 *Glomus mosseae Glomus fasciculatum*	Plots, temperate rainfed conditions	Nutrient agar medium, coalvitamin medium, potatodextrose supplemented with Rose-Bengal and streptomycin (30 g/mL)	Solarized, disinfected and natural soil plots (21% sand, 35.7% silt 43.3% clay; pH 7.4)	An increase of concentration of bacteria and/or fungal strains in the inoculated tests has been registered	Sharma et al., 2011
*Azotobacter chroococcum Azospirillum brasilense Glomus fasciculatum*	Open field	Jensen’s medium and N-free maltase medium	Soil (pH 7.12, organic carbon 9.6 g/kg)	Viable counts of the microbial population in the rhizosphere increased significantly in all the treatments over control but decreased under chemical fertilizers treatment	Singh et al., 2013

Moreover, the potential of culture-dependent methods were shown to depend also on the experimental conditions utilized. For example, Castanheira et al. (2017) were able to quantify the colonization of a consortium of bacteria with a cultivation-based approach. However, they utilized FISH and GFP-labeling, combined with confocal microscopy to follow their behavior and coexistence in the plants. Sharma et al. (2011) conducted a field experiment on apple plants using a culture-dependent approach to evaluate the microbial changes due to the application of a consortium formed by bacteria (*A. chroococcum* AZ1 and AZ2) and fungi (*G. fasciculatum* and *G. mosseae*; Table 1).

For all detection methods based on cell growth, the organism’s viability under culture conditions is essential. However, optimal culturing conditions in the lab may not be optimal for retrieving viable microorganisms from the environment: only those that can adapt from the environmental conditions to culture conditions will be possible to observe. Factors affecting the adaptation of an individual species may include lag time or the formation of cooperative clusters in spatially structures environments, or the different strategy of growth kinetics (oligotrophy, copiotrophy, and the r-K continuum; Pianka, 1970, 1972; Hengeveld et al., 2002), or interactions based on a substrate (Canfora et al., 2017).

The plate-count approach strongly underestimates the active microbial biomass. By analogy, the population sizes of bioinocula could be severely underestimated by cultivation methods (Ayrapetyan et al., 2015). Consequently, isolation in liquid cultures or on solid agar media is mainly used to monitor the potential activity, morphology, and physiological characteristics of microbial groups that perform specific functions in soil (Blagodatskaya and Kuzyakov, 2013) rather than verifying the presence of specific species or strains. However, the use of specific liquid media for isolation often results in enrichment cultures: although not yet growing in axenic culture, the target organism may tend to occur in enhanced abundance. Sequential use of the same selective liquid medium may result in a highly enriched population of target microorganisms that can subsequently be placed on solidified medium for purification (Van Kessel et al., 2015). In some cases, using solid agar media reduces the stress imposed by inter-organism competition allowing single populations to better grow out in colonies for further purification and isolation. This approach has been traditionally helpful and is still being used to recover novel isolates in pure culture (Pascual et al., 2016).

New methods have been developed to isolate new strains, which can be potentially used to track them in the soil. Cells’ encapsulation in gel microdroplets, for massively parallel microbial cultivation under low nutrient flux conditions, followed by flow cytometry to detect microdroplets containing microcolonies, allowed to detect previously uncultured strains (Zengler et al., 2002). A cost-effective and easy method that allowed on-site determination of live and dead bacterial cells’ concentration using a fiber-based spectroscopic device (optrode) was used to analyze a sample containing live and dead bacteria at varying ratios and calculate the concentration of each population (Ou et al., 2019).

Previously uncultivated microorganisms were detected using a simulated natural environment (Kaeberlein et al., 2002). A novel method of *in situ* cultivation (ichip) led to a significant increase of colony count compared to that observed on synthetic media, favoring species commonly not detected in standard Petri dishes (Nichols et al., 2010). Chaudhary and coworkers (Chaudhary et al., 2019) developed a new diffusion bioreactor to cultivate hidden bacterial communities in their natural environment and were able to recover more than 400 bacteria isolates, including 35 previously uncultured strains.

They may be present in the soil as unculturable and non-sporulating fungi, such as the endophytic and mycorrhiza forming species (Tsui et al., 2011). Indeed, he arbuscular mycorrhizal fungi (AMF) are not able to be cultivated on laboratory growth media and can only be recovered from the soil with trap plants and then counted using different methods such as the magnified intersections method (McGonigle et al., 1990), the grid-line intersect method (Newman, 1966; Marsh, 1971; Trouvelot et al., 1986), or gradient centrifugation to recover spores (Hayman, 1984; Schenck and Perez, 1990). Identification of these fungi was traditionally performed based on their spore morphology, which is time-consuming and requires considerable expertise (Pagano et al., 2016). However, monitoring of AMF can be performed by assessing root colonization. Phillips and Hayman (1970) developed an easy standard method to stain AMF in roots, which has undergone several modifications to adapt it to the roots’ characteristics from diverse plant species (e.g., Derkowska et al., 2015). Other methods based on biochemical analyses have also been proposed to monitor AMF. For example, ergosterol has been used as a biomass indicator to compare the growth of different AMF (Frey et al., 1994; Fujiyoshi et al., 2000; Hart and Reader, 2002; Lovelock et al., 2004). A method for quantification of glomalin, a fungal protein (or protein class) deposited in the soil by AMF (Rillig, 2004), which content is correlated to AMF presence (Steinberg and Rillig, 2003), based on the Bradford protein analysis (Bradford, 1976), was improved by using ELISA or SDS-page (Rillig, 2004). The analysis of AMF population size in soil, without discriminating between species, can be determined with the MPN (Most Probable Number) bioassay, which is similar to the dilution method used for culturable microorganisms (Alexander and Clark, 2016).

Most of the fungi used as biopesticides, such as the entomopathogenic species, are easily cultivated and isolated, growing optimally on standard culture media. Laboratory techniques to isolate the commercially most exploited genera of entomopathogenic fungi and bacteria from soil relying on culture-based methodologies were recently reviewed (Sharma et al., 2021) to isolate entomopathogenic fungi and bacteria from soil.

To improve the isolation of microorganisms in soil, a particular culture medium (SIA) allowing the isolation of viable but non-culturable strains within 1–3 months of incubation at 28°C could help develop different cultural methods for the diagnosis of unculturable fungi (Sia et al., 2018). However, different co-formulants could behave differently on the survival of bacteria in formulations (Omer, 2010; Vassilev et al., 2020). As alternatives, or in combination, to the classical cultivation methods, the MicroResp (Creamer et al., 2016), and community-level physiological profiles (Frąc et al., 2017) methods can be used for the identification of bacteria species. The latter method can be performed using the Biolog system (Al-Dhabaan and Bakhali, 2017) which is based on patterns of single carbon source utilization. A method based on multiple culture conditions combined with the rapid identification of bacteria (by MALDI-TOF mass spectrometry and 16S rRNA sequencing), named “culturomics,” which was developed for the identification of previously unculturable bacterial species of the human gut microbiome (Lagier et al., 2016, 2018) could be applied to isolate less abundant and unculturable microorganisms from the soil.

### DNA-Based Technologies for Tracking of Bioinoculants in Soil and Assessing Their Impact on the Microbial Community

The concept of DNA barcode as “a small piece of the genome found in a broad range of species,” is not new and has been widely used for many years across all life forms, including microorganisms, to distinguish a species from another (Chakraborty et al., 2014; Kress et al., 2015). The barcode is derived from a PCR amplicon of a target sequence used to identify (or barcoding) a microorganism distinguishing it from other species. However, DNA barcodes are not error-free, and single species barcoding needs to be designed based on robust genetic distances to obtain unique and highly discriminant markers. The genetic variability of individual strains, sometimes closely related but different in genomic traits, is exploited to discriminate the individual species but may, as well, provide inaccurate identifications (Hebert et al., 2003; Chakraborty et al., 2014). Markers based on sequences characterized amplified regions (SCAR) have been widely used as molecular probes for tracking the fate of fungi (Abbasi et al., 1999; Dauch et al., 2003). SCAR markers are based on universal primers, i.e., sequences universally present with highly conserved flanking regions, which, however, are able to discriminate only at genera or species groups levels, but not at species level within a pool of microorganisms (Barberio et al., 2001; references included in Table 2). However, markers suitable to monitor or discriminate the introduced bioinocula from native soil strains should be species-specific (Salazar et al., 2000; Grosch et al., 2007).

**TABLE 2:** List of PCR-based and culture-dependent methods: advantages and drawbacks.

Methods	Scale	Advantages	Limitations	Applicability to monitor introduce species in soil	References
PCR-Universal primers based	Whole community	Parallel analysis of high sample number	Spatial and macro scale information depending on sampling accuracy	Not	Barberio et al., 2001; Lueders and Friedrich, 2003; Kowalchuk et al., 2006; Trabelsi and Mhamdi, 2013; Canfora et al., 2014a
PCR-trait based	Selected groups/domains	Targeted gene amplification	Applicability depending on degree of knowledge of target species	Yes	Salazar et al., 2000; Rehner and Buckley, 2003; Grosch et al., 2007; Schwarzenbach et al., 2007; Babić et al., 2008; Canfora et al., 2016; Tartanus et al., 2021
Amplicon sequencing	Selected microorganisms	Targeted gene detection	Qualitative information; accuracy depending on taxonomic assignments against reference databases	Yes	Michaelsen et al., 2010; Pangallo et al., 2014
Real-time PCR (qPCR)	Whole community; selected organisms; selected groups/domains	Targeted gene amplification and quantification	Applicability depending on specific and discriminant primer pair design; not distinguish living from dead organisms	Yes	Enkerli et al., 2001; Rehner and Buckley, 2003; Schwarzenbach et al., 2007; Timmusk et al., 2009; Canfora et al., 2016, 2017; Tartanus et al., 2021
Culture-based methods	Cultivable community, ability of the microorganisms to grow under artificial conditions	Assesses living (culturable) microbes; Able to recognize viable cells in a sample; Easy to quantitate cells in a sample; High sensitivity with appropriate media	Risk of contamination; High skill level is necessary for optimal results; Time and resource intensive; Relies on phenotypic biochemical characterization	Yes	Al-Awadhi et al., 2013; Varjani et al., 2018; Nowrotek et al., 2019; Lee et al., 2021; Sharma et al., 2021; Tartanus et al., 2021
**Methods to evaluate the impact of inoculative species on soil local microbial communities**					
T-RFLP	Whole community; selected groups/domains	Discriminant bands could show a putative species, characteristic of a specific soil/treatment; PCR products can be purified and used to identify microorganisms through several tools such as the enzymatic digestion coupled with the separation of single amplicons by sequencer, the sequence analysis of excised bands, and the construction of clone libraries, facilitating a more reliable phylogenetic identification of microorganisms.	Many problems come from PCR step and primers biases: length and sequence polymorphism, choice of primers, primer specificity and degree of mismatch, significant variability of extracted eDNA (environmental DNA)	Not/suitable to monitor the impact	Michaelsen et al., 2010; Canfora et al., 2015; McKinnon et al., 2018
DGGE	Whole community; selected groups/domains	The bands reflect microbial diversity in the sample, and their relative intensity reflects abundance.	Patterns with low discriminatory power.	Not/suitable to monitor the impact	Smalla et al., 2001; Pellegrino et al., 2012; Pangallo et al., 2014
Metagenomics	Whole community	Untargeted gene screening; assessment of diversity and functional potential	A limited number of replicates; computational power required	Not/suitable to monitor the impact	Pace, 1997; Roesch et al., 2007; Morgan et al., 2010; Lombard et al., 2011; Kim et al., 2013; Canfora et al., 2014a, 2018; Zhou et al., 2015

Specificity for a target organism is a critical element in the use of any PCR-based technique. PCR-based techniques that proved to be successful in targeting single species in complex communities is focused on the natural polymorphisms of genomes. Short unique sequences allowed the use of species-specific probes to discriminate the introduced organisms in soil (Felici et al., 2008; Öpik et al., 2010; Stockinger et al., 2010; Sýkorová et al., 2012; Trabelsi and Mhamdi, 2013; Canfora et al., 2016, 2017; Malusá et al., 2016; Tartanus et al., 2021).

The possibility of targeting short sequences allowed to bypass the disadvantages of a simple PCR-based tool and the technical gap of universal primers. Simple sequence repeats have a polymorphic character that allows producing highly discriminant fingerprints (Rehner and Buckley, 2003; Schwarzenbach et al., 2007; Babić et al., 2008), showing a good discrimination power also for eukaryotic microorganisms such as filamentous fungi (Canfora et al., 2016).

Real-time quantitative PCR (qPCR) is a technique allowing quantifying genetic biomarkers at species, genera or domain-specific levels through the detection of the fluorescence produced by a reporter molecule increases linearly as the PCR cycles proceed in real time. In this framework, quantitative PCR-based techniques offer some opportunities for monitoring single species persistence or functions across the soil communities’ complexity (Schwieger and Tebbe, 2000; Hirsch et al., 2010; Han et al., 2012; Trabelsi and Mhamdi, 2013).

The fluorescence detected is directly proportional to the amount of labeled DNA template present in the PCR (Wen et al., 2009; Blaya et al., 2016). Fluorescent reporters include dyes (i.e., SYBR green) that intercalate with any (non-specific) double-stranded DNA or sequence-specific DNA probe (TaqMan probe), allowing the detection, after hybridization, of the probe with its complementary sequence. The preparation of the qPCR standard and the calibration of the qPCR assay are the two critical steps of the method, affecting the occurrence of false-positive results. qPCR benefits of direct DNA extraction followed by PCR amplification in real-time; this approach is faster than simple PCR and, when coupled with trait-based PCR primers, unique for the microorganisms of interest, allows the simultaneous detection and quantification of the strain.

**FIGURE 5:**
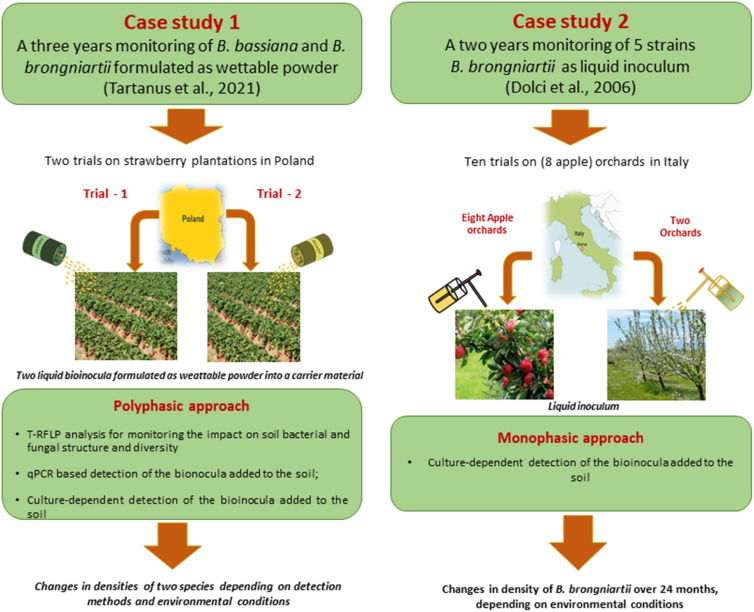
Two case studies of different methods for detection and monitoring of inoculum persistence.

Several examples in the literature reported that using Real-Time PCR coupled with trait-based PCR permit the detection of the introduced species, simultaneously evaluating their relative abundance within the microbial community (Clesceri et al., 1999; Babić et al., 2008; Couillerot et al., 2010; Paulo et al., 2014; Canfora et al., 2016, 2017; Mandakovic et al., 2018; Tartanus et al., 2021). However, the suitability of a polyphasic approach based on DNA-based methods emerged clearly from a 3-year long study on the persistence of two entomopathogenic fungi (*Beauveria brongniartii* and *B. bassiana*) on two sites (Canfora et al., 2016; Tartanus et al., 2021). The abundance of the fungi assessed by mean of a culture-dependent approach, resulted to be lower compared to culture-independent methods. It is noteworthy that previous studies focusing on the detection of the same species by using culture-dependent methods were still able to demonstrate the survival and the establishment of *B. brongniartii* over 24 months (Dolci et al., 2006) or 7 years after its application (Enkerli et al., 2004; Figure 5).

The Real-Time PCR can be successfully used to determine functional groups and have a great potential to estimate the real activity status of certain microorganisms by quantifying relevant functional genes. Although qPCR-real time can be helpful for monitoring and directly quantifying bacteria or fungi introduced in the soil, it needs primer pairs specifically designed to obtain a highly discriminating amplicon. A summary of the major advantages and drawbacks of PCR-based methods is presented in Table 2.

In recent years, increasing efforts have been devoted to improve the knowledge of species-specific marker gene sequences (Yilmaz et al., 2011) or the species genome sequence (Field et al., 2008). The whole genome of a microbial inoculant can be used to design qPCR primers and probes based on the core genome sequences that do not match with other sequences present on BLAST (Rillig et al., 2019). Alternatively, it is possible to exploit the 16S or 18S sequence for bacterial or fungi, respectively, to design specific qPCR primers. Irrespective of the species concerned, there is a clear need for a broader knowledge of microorganisms’ genome sequence that could also be exploited for monitoring of the strains used in microbial inoculants formulations. This approach is currently developed in the work of the project Excalibur^1^.

Different methods based on the analysis of genetic polymorphisms (T-RFLP; DGGE) have been extensively used to study complex microbial communities in diverse soil ecosystems and environments but proved to be unable to discriminate single target species (Schwieger and Tebbe, 2000; Hirsch et al., 2010; Han et al., 2012; Pellegrino et al., 2012; Trabelsi and Mhamdi, 2013). Nevertheless, they resulted suitable to evaluate the impact of bioinocula on autochthonous microbial communities’ structure and diversity (Michaelsen et al., 2010; Canfora et al., 2015). For example, T-RFLP analysis, which is based on the detection of a single restriction fragment within conserved genomic sequences, amplified directly from the soil DNA is capable of surveying dominant members within the microbial population that comprise at least 1% of the entire community. The combination of PCR, restriction enzyme digestion and electrophoresis on an automated sequencer produces patterns of terminal restriction fragments that can be used to examine microbial community structure and community dynamics in response to bioinocula application (Gao et al., 2012; Trabelsi et al., 2012) or changes in environmental conditions (Mengoni et al., 2006) as well as to study microbial population composition in natural habitats (Canfora et al., 2015). The fingerprint of soil can be obtained by DGGE analysis under DGGE, which enable the separation of DNA fragments of identical length but different sequence (Pellegrino et al., 2012; Pangallo et al., 2014). T-RFLP and DGGE are capable to detect 95–99% of the microbial community, and biomarkers can be derived from sequences present on 16S or 18S or 23s rRNA genes, or ribosomal ITS regions, in the case of bacteria, archaea or fungi, respectively. The presence of discriminant bands could indicate putative species that may be characteristic of the soil sample; however, only cloning and sequencing of the DNA band can confirm the result.

In the last decade, metagenomic methods have been increasingly applied to characterize microbial communities or target groups of microorganisms in different soil ecosystems and environmental compartments (Mocali and Benedetti, 2010; Nesme et al., 2016; Jansson and Hofmockel, 2018; Taş et al., 2021). Sequencing the whole DNA [NGS, third-generation sequencing (TGS) techniques] isolated from a specific soil environment allows to evaluate the total genomic diversity, which is a different concept compared to total species diversity assessed with conventional molecular methods. The metagenomic approach has been applied to study the structure and composition of uncultivated and unculturable microbial populations in specific ecosystems in response to selective pressures and spatio-temporal factors (Daniel, 2005; Fierer et al., 2013; Howe et al., 2014; Canfora et al., 2014b; Feng et al., 2018). Bharti and Grimm (2021) reviewed the sequencing technologies used in the last 25 years, trying to list challenges and best practice protocols using sequencing-based technologies for microbiome analysis. A drawback of the NGS method lies in the possibility of generating overlapped short-read sequences since the DNA is partially digested with enzymes into lengths that are readily sequenced. In this framework, TGS platform like PacBio (Pacific Biosciences RS II/Sequel) and Oxford Nanopore MinION sequencing technologies are attracting the attention of the molecular ecologist showing promising results since they produce very long reads (from 1 to 100 kb; Santos et al., 2020; Bharti and Grimm, 2021). However, despite the recent output improvement of Sequel from ∼0.5–1 to ∼5–10 Gb, the application remains scarce. The Illumina sequencing platform produces high read counts. However, computational power and intensive time-consuming work is required to assign taxonomic identity due to the large and multivariate data structure. The sequences can be assembled to recreate the entire sequence of targeted microorganisms’ genomes in soil, but the results cannot be used as specific and discriminant characters for single microbial inoculants. Moreover, the estimation of microbial diversity obtained with the analysis depends on the resolution and length of the sequence of the rRNA gene marker used and the quality of sequenced libraries, the computational bioinformatics parameters used and the whole process workflow. On the other side, long reads sequence allows identifying rare genomes inaccessible compared to the short-reads platform. However, both sequencing generations platform suffer from many biases in sample collection, nucleic acid extraction, experimental errors and bioinformatic analysis. In the last years, modern sequencing technologies have taken over the Sanger classical technologies for metagenomic profiling of microbial communities. Specific genetic markers (Shen et al., 2021) used to identify target microbes from several environments (63F-1087r of 16S rRNA gene; ITS1-4 or ITS2-4, β-tubulin, calmodulin gene) with the classical Sanger approach do not overlap with standardised barcodes (V3-V4 or V3-V4 or V7-V8 for 16S rRNA, ITS1f- ITS2) used to build an international collection of sequences derived from the taxonomical classification of the communities with NGS platform (Cristescu, 2014). This generates an increasing gap between classical morphological and DNA-based identification (Cristescu, 2014). Owing to this gap, the identification process consisting of taxonomic assignment based on the alignment of sequences with international database (NCBI) suffers from errors that can influence microbial diversity estimates. A robust taxonomic inference can help us calibrate microbial diversity estimates and prevent erroneous interpretations. It easy to imagine that if we start from a good knowledge based on morphological, physiological, or functional traits, we will likely able to generate specie-specific barcode. Overall, we regard metabarcoding of complex bacterial and fungal communities as a highly promising approach to the discovery of patterns of complex communities.

## Future Perspectives and Conclusion

The prospect of manipulating crop microbial populations by inoculating beneficial bacteria and fungi to increase plant growth has shown considerable aptitude in laboratory and greenhouse studies; however, responses have been variable in the field (e.g., Arora, 2015; Kaminsky et al., 2019; Malusá et al., 2020). Recent progress in understanding the biological interactions between the practical requirements for microbial inoculant formulation and delivery systems will increase this technology’s evenness in the field and facilitate its commercial development.

The use of multi-strain inocula of microorganisms with known functions and bioinocula with different desirable traits and tolerance to environmental conditions (Ahmad et al., 2008; Vassileva et al., 2020) may increase the consistency of results in the field. Nevertheless, tracking and monitoring the fate and metabolic activity of microbial inoculants and their impact on rhizosphere and soil microbial communities are needed to assure their safe and trustworthy application to crops. A combination of classical and molecular techniques should be used to monitor any inoculant’s strain after its release into the soil. Both culture-based and culture-independent approaches have their specific advantages and limitations. Appropriate metabolic and physiological characterization of the microorganisms used for the formulation of bioinoculants would bridge the culture-independent and the culture-dependent methods. The chance of designing optimized and custom specific probes with high specificity increases when a proper identification and genetic characterization of the species involved in the formulation of a soil microbial inoculant is available. This strongly contributes to the success of detection and monitoring analysis that also depends on a proper sampling method which shall assure the quality and the robustness of the data generated.

The genetic or functional characterization of the inoculant allows to fully exploit the potential of PCR-based methods that tackle species-specific functional traits or genome polymorphism, increasing the power of sensibility, specificity and sensitivity of the technique. However, although many available methods can be coupled to address the request of a discriminant and specific detection tool to monitor introduced bioinocula strains in soil, it is generally acknowledged that accurate and standards methods are yet to be developed. An impetus toward this goal could derive from the field of microfluidics applications and tools like the “lab-on-chip” (Aleklett et al., 2018) could have the potential to address some of the major challenges of tracking and monitoring of bioinoculants. The “lab-on-chip” (microarray) technology opens the avenue for the parallel, high-throughput multiple identification of target microorganisms and genes in environmental samples (Wilson et al., 2002; Chandler et al., 2010; Schreiner et al., 2010; Mocali et al., 2020). Data obtained with amplification microarrays correlated well with conventional small subunit rRNA qPCR results (Chandler et al., 2010). Microarrays would also evaluate bioinocula functional activity; in the case of expressed genes, they represent one of the most powerful approaches due to their unique ability to query the mRNA expression. Environmental DNA barcoding can be used to monitor the impacts of introduced species on soil autochthonous microbial diversity, evaluating the sensitivity of vulnerable microbial native species, and better identify their distribution. However, it is not valuable for species-specific tracking.

For all these reasons, a polyphasic approach, comprising the use of different techniques, would be most practical and suitable for monitoring microbial inoculants in soil. This approach would allow fine-tuning the application method for different kinds of bioinocula to better foster the efficacy of the bioinocula, to better assess the bioinocula impact on native microbial communities, finally, resulting in an increased practical application of biofertilisers and biopesticides.

## Author Contributions

LC and AM: conceptualization. AM, LC, FPi, CC, and FPa: methodology. LC, CC, and FPa: formal analysis. LC, AM, FPi, EM, and SM: writing—original draft preparation. EM, LC, AM, and FPi: writing—review and editing. All authors have read and agreed to the published version of the manuscript.

## Conflict of Interest

The authors declare that the research was conducted in the absence of any commercial or financial relationships that could be construed as a potential conflict of interest.

## Publisher’s Note

All claims expressed in this article are solely those of the authors and do not necessarily represent those of their affiliated organizations, or those of the publisher, the editors and the reviewers. Any product that may be evaluated in this article, or claim that may be made by its manufacturer, is not guaranteed or endorsed by the publisher.

## Footnotes

1
www.excaliburproject.eu

2
https://www.arb-silva.de/fish-probes/

## Supplementary Material

Supplementary Table 1 EndNote file.
